# TheViral MetaGenome Annotation Pipeline(VMGAP):an automated tool for the functional annotation of viral Metagenomic shotgun sequencing data

**DOI:** 10.4056/sigs.1694706

**Published:** 2011-06-30

**Authors:** Hernan A. Lorenzi, Jeff Hoover, Jason Inman, Todd Safford, Sean Murphy, Leonid Kagan, Shannon J. Williamson

**Affiliations:** 1 J.CraigVenterInstitute,9704 Medical Center Drive, Rockville, MD, 20850, USA; 2 J.CraigVenterInstitute,10355 Science Center Drive, San Diego, CA 92121, USA

**Keywords:** J. Craig Venter Institute, metagenomic annotation, viral annotation

## Abstract

In the past few years, the field of metagenomics has been growing at an accelerated pace, particularly in response to advancements in new sequencing technologies. The large volume of sequence data from novel organisms generated by metagenomic projects has triggered the development of specialized databases and tools focused on particular groups of organisms or data types. Here we describe a pipeline for the functional annotation of viral metagenomic sequence data. The Viral MetaGenome Annotation Pipeline (VMGAP) pipeline takes advantage of a number of specialized databases, such as collections of mobile genetic elements and environmental metagenomes to improve the classification and functional prediction of viral gene products. The pipeline assigns a functional term to each predicted protein sequence following a suite of comprehensive analyses whose results are ranked according to a priority rules hierarchy. Additional annotation is provided in the form of enzyme commission (EC) numbers, GO/MeGO terms and Hidden Markov Models together with supporting evidence.

## Introduction

Viruses are the most abundant biological agents and comprise the majority of the biodiversity on Earth [1-3]. However, understanding the population biology and dynamics of viral communities in the environment is difficult because their hosts (predominantly microbes) are unknown and cannot be grown in culture. Furthermore, the study of viral diversity is hampered by the lack of a universally conserved gene across all viral species, analogous to rDNA genes in cellular organisms. Metagenomic shotgun sequence analysis of viral communities helps to alleviate these constraints and is currently the most widely used approach to study the biodiversity of viral populations isolated directly from the environment.

The recent development of faster and cheaper next generation sequencing technologies has contributed to an exponential growth of metagenomic sequencing data, transforming our view of the microbial world. Despite the advancements in sequencing technology, functional annotation of metagenomic sequences is still very challenging. Metagenomic data originate from heterogeneous microbial communities, are usually noisy and partial, and reads frequently contain truncated open reading frames (ORFs). Complicating this landscape, the vast majority of viruses isolated from environmental samples are novel and consequently most of their genes do not have homologous sequences in the public databases, making functional annotation even more difficult.

Currently, there are a number of publicly available bioinformatics tools for the taxonomic (Ribosomal Database Project(RDP) [4],Greengenes [5],MEGAN [6], pplacer [7]) and functional (IMG/M [8], CAMERA [9], MG-RAST [10]) analysis of metagenomes. While IMG/M facilitates the functional analysis of pre-selected metagenomic data, it does not support the input and analysis of external user data. CAMERA allows for the construction of customized workflows for the analysis of external metagenomic data including functional annotation using RAMMCAP based on PFAM, TIGRFAM and COGs. MG-RAST is an alternative web-resource that performs metabolic reconstructions using SEED subsystems [11] and builds automated phylogenetic profiles of metagenomic data provided by the scientific community. While MG-RAST has been used for the functional annotation of multiple viral metagenomes [12], it is not ideal for the characterization of viral metagenomic data since functional classification is solely dependent on similarity to FIGfams [13], protein families developed from manually curated bacterial and archaeal proteins. Another limitation of this tool is that it does not search for conserved protein domains or motifs that could provide additional clues about the functional roles of genes present in metagenomic samples.

Here we describe a viral metagenomic annotation pipeline (VMGAP) that is currently utilized at the J. Craig Venter Institute (JCVI) for the functional annotation of viral metagenomic datasets. This pipeline incorporates a number of HMM and PSSM searches and makes use of a suite of specialized databases to improve the functional identification of viral genes. Results can be imported into JCVI Metagenomic Reports (METAREP) [14], an open source tool for high performance comparative metagenomics that allows users to view, query, browse and compare extremely large annotated metagenomic data sets.

## Requirements

The VMGAP requires a protein multi fasta file as input and the local installation of several open source programs, packages and public databases. The required software and packages are HMMER [15], NCBI-toolkit (blast searches [16]), SignalP (signal peptide prediction [17]) [18]and TMHMM [19,20] and PRIAM (Ecnumber prediction [21]) [22]. Among the public databases searched by the pipeline are GenBank NRDB, GenBank environmental databases ENV_NT and ENV_NR, UniProtDB [23], OMNIOMEDB [24], PFAM [25] and TIGRFAM [26] HMMDBs, ACLAME protein and HMMDBs [27],GenBank CDDDB [28] and pfam2gomappingsDB [11].

## Procedure

**Figure 1:**
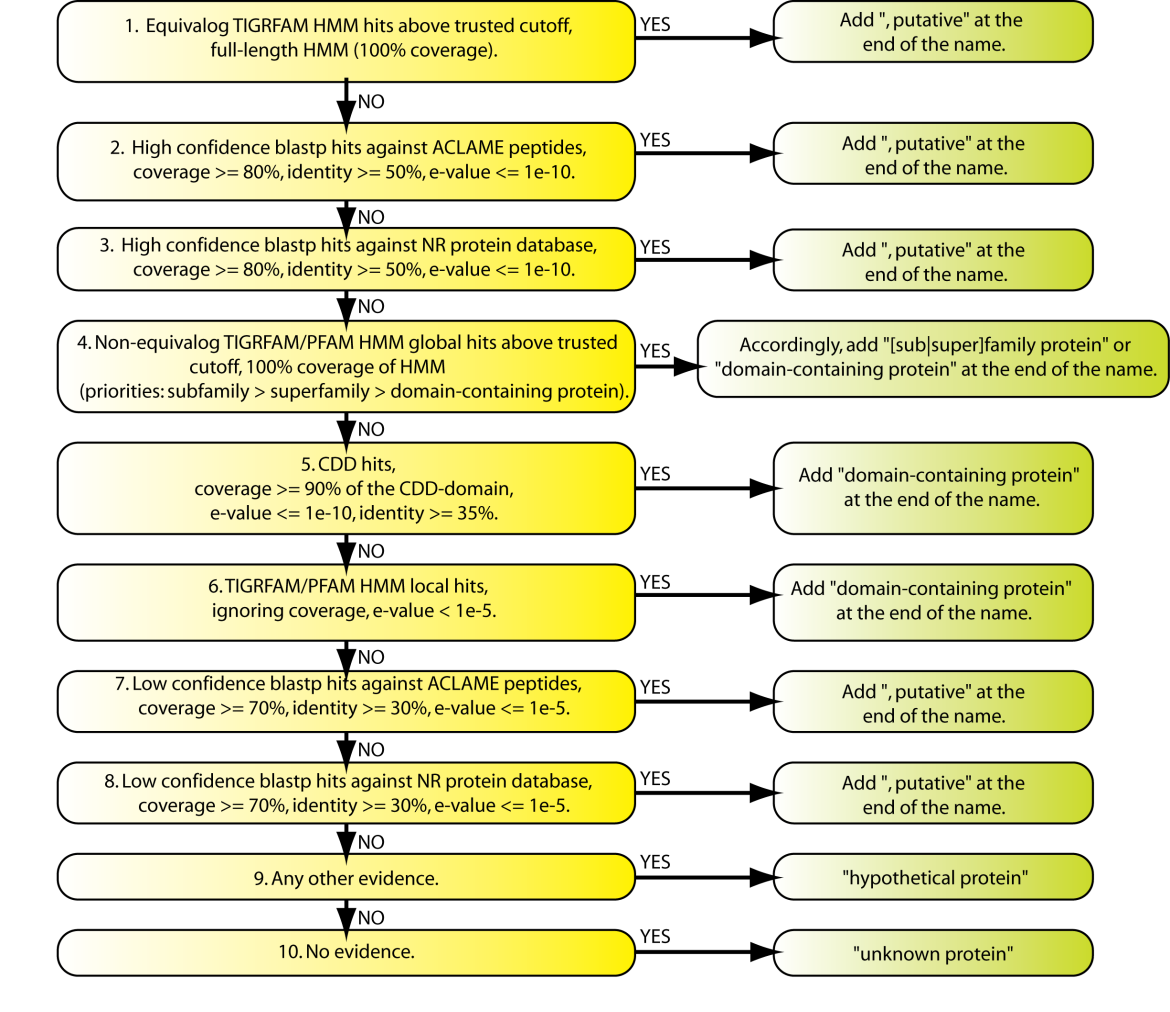
Naming rules used for functional annotation of the VMGAP.

The JCVI VMGAP consists of two consecutive steps: (1) database searches and (2) functional assignments. The pipeline uses as input a multifasta file containing the translations of all open reading frames (ORFs) predicted in a metagenomic sample. Protein coding genes are predicted using the structural annotation pipeline [29], that is based on a combination of naïve 6-frame translations and MetaGeneAnnotator [30,31], an *ab initio* gene finder program that uses empirical data including sequence-based composition, distance and orientation of genes of completely sequenced genomes to identify protein coding genes. Once uploaded, protein sequences are used to query several databases to identify protein features and similarities as schematically represented in Figure 1. During step 1, the VMGAP performs the following sequence similarity searches:

### 1) Blastp searches against a non-redundant protein database

The non-redundant protein database encompasses several public protein databases (GenBank NR, UniProt, PIR and OMNIOME) where each set of redundant peptides are condensed into a single database entry without losing useful information recorded in the fasta headers, such as EC numbers, product names, and taxon identification number. The VMGAP reports the top 50 hits with e-values ≤1x10^-5^.

### 2) Blastp searches against the ACLAME database

ACLAME is a public protein database of mobile genetic elements (MGEs), including bacteriophages, transposons and plasmids [27]. Proteins are organized into families based on their function and sequence similarity, and families of 4 or more members are manually annotated with functional assignments using GO and MeGO terms (an ontology dedicated to MGEs developed by ACLAME). All blastp hits with e-values ≤1x10^-5^ are reported.

### 3) Blastp and tblastn searches against environmental protein databases

The VMGAP queries three different environmental composite databases at the amino acid level: (i) ENV_NR, a GenBank non-redundant protein database that includes many environmental datasets, (ii) an in-house database (SANGER_PEP) composed of proteins coded by Sanger-based viral metagenomic samples not represented in ENV_NR (**Table 1**), and (iii) ENV_NT, a collection of nucleotide sequences from metagenomic datasets deposited in GenBank. The purpose of these analyses is to determine how similar the viruses are within the query metagenomic samples to viruses and microbes that inhabit the different environments represented in the subject databases. The VMGAP reports all blast hits with e-values ≤1x10^-3^.

**Table 1:** Metagenomic libraries incorporated into the Sanger environmental protein database

**Library Name**	**Reference**
Viral metagenomes from Yellowstone hot springs (Bear Paw)	[32]
RNA viral community in human feces	[33]
viral metagenomes from yellowstone hot springs (Octopus)	[32]
Virus from Human Blood	[34]
Virus from Human Feces	[35]
Virus from Marine Sediments	[36]
Uncultured marine viral communities (Mission Bay)	[37]
Uncultured marine viral communities (Scripps Pier)	[37]
Coastal RNA virus communities	[38]
Chesapeake Bay virioplankton	[39]
Virus from equine feces	[40]

### 4) HMM searches against PFAM/TIGRFAM and ACLAME HMM

In addition to similarity searches against protein databases, the VMGAP looks for the presence of HMMs from two databases, PFAM/TIGRFAM (a database of HMMs representing conserved protein domains) and ACLAME-HMMs (a compilation of HMMs that describe each of the protein families found in ACLAME). PFAM/TIGRFAM HMM searches are carried out in two different ways, either requiring a global or local alignment to the HMMs. Local HMM alignments increase sensitivity in the detection of conserved protein domains, particularly when the predicted peptide is truncated and extends to the end of the read, which is noted frequently in metagenomic datasets. All HMM hit with e-values ≤l1x10^-5^ are recorded for further analysis.

### 5) RPS-Blast against NCBI CDD database

The NCBI Conserved Domain Database (CDD) database is a collection of position specific scoring matrices representing conserved protein domains, protein families and superfamilies compiled from NCBI-curated domains [41], PFAM/TIGRFAM, SMART [42] and COG [43]. In spite of the overlap, PSSMs derived from PFAM/TIGRFAM do not behave exactly the same as their HMM counterparts, and in some cases these searches can identify domains where HMMs fail. The VMGAP stores all hits with e-values ≤ 1x10^-5^.

### 6) Identification of transmembrane domains and signal peptides

To discover transmembrane proteins and signal peptides that could be associated with the surface of viral particles, the VMGAP utilizes two programs, SignalP for the identification of signal peptides, and TMHMM, a program that detects candidate transmembrane domains.

### 7) Assignment of EC numbers

To aid in the metabolic reconstruction of metagenomes, the VMGAP makes use of PRIAM, a collection of PSSMs where each matrix represents an enzymatic function and is assigned to a particular EC number. Metagenomic samples are scanned for the presence of these PSSMs with RPS-Blast recording only those hits with e-values ≤1x10^-10^.

### 8) Rules hierarchy

Functional assignments of predicted peptides are carried out by retrieving the functional information produced from the results of the analyses performed in the previous steps following a series of pre-defined rules (Figure 1). Rules prioritize the use of a certain piece of evidence over another based on how informative, trustful and accurate that evidence is. As shown in Figure 1, hits against equivalog TIGRFAM HMMs [26]are the highest ranked supporting evidence for functional assignments in the VMGAP. Therefore, any protein that hits above the trusted cutoff of one entire copy (100% coverage with respect to the length of the HMM) of an equivalog TIGRFAM will automatically inherit the functional annotation associated to that particular HMM. The second and third tiers of evidence are constituted by highly significant BLASTP hits against ACLAME DB and the non-redundant protein database respectively; having at least 80% coverage (with respect to the shortest sequence), 50% identity and an e-value ≤ 1x10^-10^. Although proteins from ACLAME DB are also included in the non-redundant protein database, entries in the former have a higher priority since they are curated and therefore provide better functional annotation. Hits against HMMs describing ACLAME protein families and PFAM/non-equivalog TIGRFAM HMMs comprise the 4^th^ and 5^th^ layers of functional evidence, giving higher priority to those HMMs representing protein families against those describing conserved protein domains. Ranked 6^th^ and 7^th^ in the rule list are respectively RPS-BLAST hits with at least 90% coverage, percent identity ≥ 35% and e-value ≤ 1x10^-10^ against NCBI-CDD profiles and local-local hits against PFAM/TIGRFAM HMMs with e-values ≤ 1x10^-5^. Finally, low-confidence BLASTP hits with at least 70% coverage, percent identity ≥ 30% and e-value ≤ 1x10^-5^ against ACLAME DB and the non-redundant protein database occupy tiers 8 and 9 in the priority list respectively. Proteins that lack the evidence types described above, but still contain some other evidence such as hits against the environmental DBs are named “hypothetical protein”. Otherwise, proteins are labeled as “unknown protein”.

## Implementation

**Figure 2:**
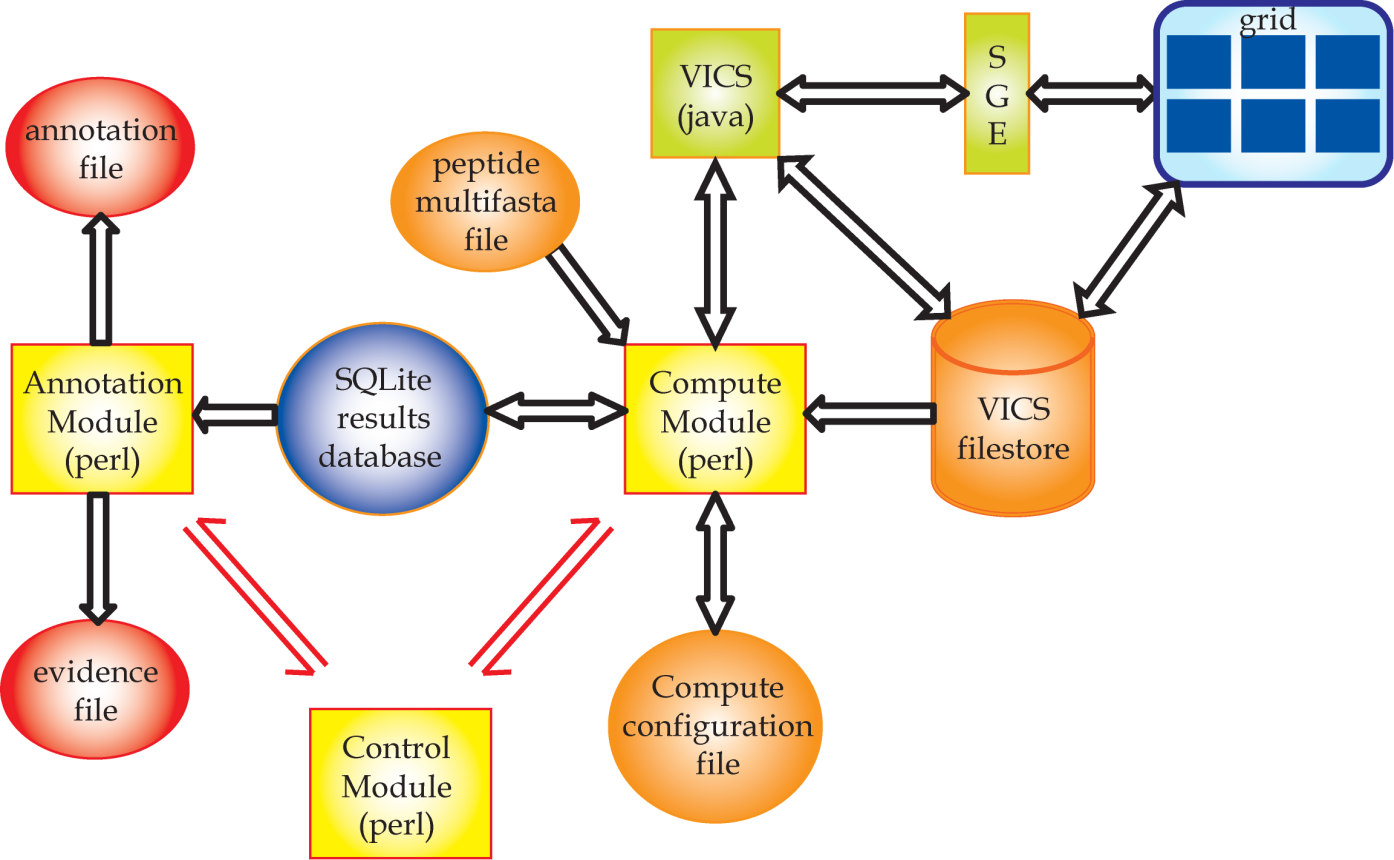
Schematic representation of the implementation of the VMGAP. The three main modules of the pipeline are depicted by yellow squares. Orange and red circles represent input and output files respectively. VICS stands for Venter Institute Compute Services; SGE stands for Sun Grid Engine job scheduler. Single and double-headed arrows indicate information flowing in one or both directions respectively.

The VMGAP consists of three major modules implemented in Perl (Figure 2): (i) the control module, which initializes the pipeline, creates a sqlite DB [44] to store the status of computations and their results, coordinates the other modules, and allows interrupted pipelines to be resumed from the point of interruption, (ii) the compute module, which tracks the status of the individual computations and loads completed computations into the sqlite database, and (iii) the annotation module, which reads the computational results from the sqlite DB and applies a set of predefined rules to generate a tab-delimited annotation file containing the final annotation for each peptide (e.g. EC/GO assignments and protein names), and a tab-delimited evidence file that stores all the evidence that supports the annotation. Each line in the annotation file contains the functional annotation for an individual peptide, while in the evidence file each line represents one particular evidence for a single protein (Table 2 and Table 3). Additionally, the VMGAP contains an optional module, also implemented in Perl, called Com2GO (Common-Name-to-Go Mappings). Com2GO can be run after the annotation module to attempt to classify the protein names using the GO hierarchy.

**Table 2:** Description of the contents of the evidence file generated by the VMGAP

1	2	3	4	5	6	7	8	9
ID	CDD_RPS	Subject definition	% cov	% ident	e-value		% ident	
ID	ALL GROUP_PEP	Subject ID	Subject definition	Query length	Subject length	% cov	% ident	e-value
ID	ACLAME_PEP	Subject ID	Subject definition	Query length	Subject length	% cov	% ident	e-value
ID	SANGER_PEP	Subject ID	Subject definition	Query length	Subject length	% cov	% ident	e-value
ID	ENV_NT	Subject ID	Subject definition	Query length	Subject length	% cov	% ident	e-value
ID	ENV_NR	Subject ID	Subject definition	Query length	Subject length	% cov	HMM description	e-value
ID	FRAG_HMM	HMM begin	HMM end	% cov	Total e-value	HMM accession	HMM description	HMM length
ID	PFAM/TIGRFAM_HMM	HMM begin	HMM end	% cov	Total e-value	HMM accession		HMM length
ID	PRIAM	EC Number	e-value				HMM description	
ID	ACLAME_HMM	HMM begin	HMM end	% cov	Total e-value	HMM accession		HMM length
ID	PEPSTATS	Molecular weight	Isoelectric point					
ID	TMHMM	Number predicted helixes						
ID	SIGNALP	signal pep	cleavage site position					

Fields 1 and 2 correspond to the protein identifier and a flag specific for each analysis, respectively. % cov, percent coverage; % ident, percent identity; CDD_RPS, RPS-Blast vs. CDD DB; ALLGROUP_PEP, Blastp vs. protein NR DB; ACLAME_PEP, Blastp vs. ACLAME protein DB; SANGER_PEP, Blastp vs. in-house viral metagenomic DB; ENV_NT, Tblastn vs. ENV_NT DB; ENV_NR, Blastp vs. ENV_NR DB; FRAG_HMM, HMM searches vs. local PFAM/TIGRFAM HMM DB; PFAM/TIGRFAM_HMM, HMM searches vs. global PFAM/TIGRFAM HMM DB; PRIAM, RPS-Blast vs. PRIAM profile DB; ACLAME_HMM, HMM searches vs. global ACLAME HMM DB; PEPSTATS, peptide statistics; TMHMM, transmembrane domain searches; SIGNALP, signal peptide searches.

**Table 3:** Explanation of the annotation file generated by the VMGAP

Column	Description	Example
1	Unique peptide ID	JCVI_PEP_metagenomic.orf.112038372243 2.1
2	Protein common name tag	common_name
3	Functional description (s)	phosphonate C-P lyase system protein PhnL, putative
4	Source of functional description assignment	AllGroup High:rf|YP_001889651.1
5	GO tag	GO
6	Gene Ontology ID (s)	go:0016887||go:0005524
7	Source of Gene Ontology assignment	PF00005||PF00005
8	EC tag	EC
9	Enzyme Commission number ID (s)	3.6.3.28
10	Source of Enzyme Commission ID	PRIAM
11	Hits against ENV_NT DB tag	ENV_NT
12	ENV_NT DB libraries hit with e-values ≤1 × 10^-3^	Hydrothermal vent metagenome FOSS10958.y2, whole genome shotgun sequence || Lake Washington Formate SIP Enrichment Freshwater Metagenome || Human Gut Metagenome (healthy human sample In-M, Infant, Female)
13	Best hit e-value per environmental library	6.65676e-54 || 2.14066e-44 || 1.34265e-46
14	Number of hits with e-value ≤1 × 10^-3^ per environmental DB library	1 || 1 || 4
15	HMM DB tag	PFAM/TIGRFAM_HMM
16	PFAM/TIGRFAM HMM hit above trusted cutoff	PF000005
17	Signalp tag	SIGNALP
18	Presence (Y) or absence (N) of predicted signal peptide	Y
19	Cleavage site position	16
20	Transmembrane domain tag	TMHMM
21	Number of predicted transmembrane domains	2
22	Protein statistics tag	PEPSTATS
23	Molecular weight	17369.86
24	Isoelectric point	9.9423

Each lane contains the annotation for a single predicted peptide. Multiple values within a field are separated by the symbol “||”.

The heart of the VMGAP is the compute module (Figure 2). This module accepts a compute configuration file (see Table 4 for the current configuration) and a sqlite results database. It compares the computations specified in the configuration with the results loaded into the sqlite results database. Missing computations are initiated, stale computations (outdated reference dataset or obsolete program options) are refreshed, and interrupted computations are resumed. The computations themselves are executed either in a local machine (for jobs that are not very computational intensive such as SignalP), or through the JCVI high-throughput computing platform named VICS web-services. VICS is a J2EE server backed by a 1600 node SGE-grid and a 2 Terabyte scratch-disk. All of the computations are started (or restarted) and then the compute module waits for them to complete. As a computation is completed, its results are parsed and loaded into the sqlite database and the status of the computation is updated. When all computations have completed, the module exits and allows the controller to proceed. The module may be interrupted manually and restarted at a later time.

**Table 4:** List of programs and parameters in the VMGAP

Pipeline job name	Program	Parameters
A CLAME_HMM	hmmpfam	E 0.001
A CLAME_PEP	Blastp	b 50 -e 1e-5
ALLGROUP_PEP	Blastp	b 50 -e 1e-5
CDD_RPS	Rpsblast	b 50 -e 1e-3
ENV_NR	blastp	b 50 -e 1e-3
ENV_NT	tblastn	b 50 –e 1e-3
FRAG_HMM	hmmpfam	E 0.001
PEPSTATS	Pepstats	None
PFAM/TIGRFAM_HMM	Hmmpfam	E 0.001
PRIAM	Priam	e 1e-10
SANGER_PEP	Blastp	b 50 -e 1e-5
SIGNALP	Signalp	t gram- -trunc 70
TMHMM	tmhmm	None

## Data Visualization and Analysis

Small files can be easily imported and analyzed in Excel. For extremely large files (more than a million entries), we recommend users to import the data into METAREP [14] for further analysis and visualization. The METAREP tab-delimited import format specifies many common annotation data types including those computed by VMGAP. To import VMGAP annotations, we recommend the mapping outlined in (Table 5). To import the data, users have to install a local version of METAREP. The source code can be found at the METAPREP website [45]. The code also contains a Perl based utility for importing METAREP tab-delimited files. Details about the installation and import process can be found in the METAREP manual which can be downloaded from the METAPREP dashboard [46].

**Table 5:** VMGAP to METAREP mapping

**METAREP input file**			**VGMAP**
Column	Field ID	Description	Description
1	peptide_ID	Unique peptide ID	Unique peptide ID
2	Library_ID	Library ID	Library ID
3	com_name	Functional description (s)	Common Names(s)
4	com_name_src	Source of functional description assignment	^a^NRdb BLAST + ^b^FRAGHMM + PFAM + TIGRFAM + PRIAM + CDD +ACLAME
5	go_id	Gene Ontology ID (s)	Gene Ontology ID (s)
6	go_src	Source of Gene Ontology assignment	PFAM + TIGRFAM + ACLAME +Com2GO
7	ec_id	Enzyme Commission ID (s)	Enzyme Commission ID (s)
8	ec_src	Source of Enzyme Commission ID	TIGRFAM + PFAM + PRIAM
9	hmm_id	Hidden Markov Model hits	ACLAME, PFAM, TIGRFAM
10	blast_taxon	NCBI taxonomy ID	Best Blast Hit NRdb
11	blast_evalue	BLAST E-Value	Best Blast Hit NRdb
12	blast_pid	BLAST percent Identity	Best Blast Hit NRdb
13	blast_cov	BLAST sequence coverage of shortest sequence	Best Blast Hit NRdb
14	filter	Any filter tag (categorical variable)	N/A

^a^JCVI non-redundant protein database; b, PFAM/TIGRFAM local-local alignment HMM database; c, data not available.

## Discussion

In recent years and with the advancement of next generation sequencing platforms, metagenomic studies have become more affordable to the scientific community. This has triggered an exponential growth in the amount of metagenomic sequencing data available within public repositories and stresses the necessity for specialized highly efficient computational tools to cope with the functional annotation of these massive datasets. There are currently a variety of metagenomic annotation tools that are available to the general public through the web. Among the most popular resources is MG-RAST, an annotation tool that offers many advantages to the user: (i) it does not require a high-throughput computer facility, (ii) it uses reads instead of proteins as input and therefore there is no need for gene predictions, and (iii) the results are classified into functional categories facilitating the analysis of data. Perhaps most importantly, the functional distributions can be compared against other datasets that were annotated with MG-RAST.

**Figure 3:**
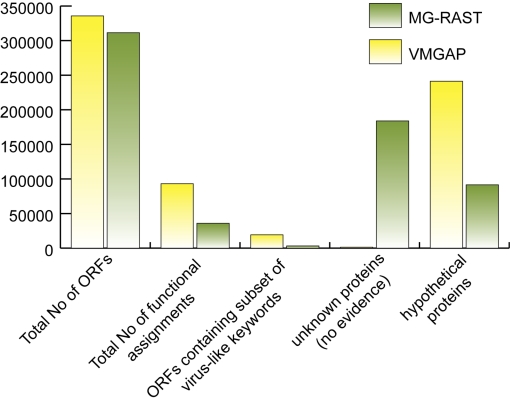
Comparative analysis of the functional annotation performance for viral libraries of the VMGAP compared with MG-RAST. Total number of functional assignments represents the amount of peptides from the viral library that gets a name other than “hypothetical protein” or “unknown” (VMGAP) or that does not have a significant hit against any FIGfam (MG-RAST). Unknown proteins are those that do not receive any evidence as described in Figure 1 (VMGAP) or that do not hit any FIGfams (MG-RAST). The following are examples of virus-like keywords used in this analysis: integrase, terminase, polymerase, recombinase, (endo|exo) nuclease, phage, viral, capsid, envelope, filament, and basal plate.

While MG-RAST is capable of providing meaningful taxonomic and functional annotation of microbial metagenomes, it is limited in its capacity to annotate viral metagenomes due to its inherent dependence of FIGfams. In order to quantitatively assess the utility of VMGAP for the functional annotation of viral metagenomic data, we ran an identical set of ~300,000 peptide sequences from a marine viral metagenomic library or their respective coding ORFs through the VMGAP and MG-RAST respectively. Analysis of the results showed that the VMGAP could assign functions to almost 16% more sequences compared to MG-RAST (names other than hypothetical or unknown, Figure 3). More specifically, when looking for viral-like enzymatic functions (e.g. integrase, endonuclease, DNA polymerase) or names describing viral-like structural functions (e.g. capsid, tail, neck, envelope), the VMGAP assigned almost 16,000 more viral-like names compared to MG-RAST. Of the sequences that received no functional names, ~72% contained some other evidence such as hits against environmental databases, PFAM domains or signal peptides while only 29% of such sequences are reported in MG-RAST (Figure3). A more in-depth analysis showed that the increase in assigned VMGAP-associated functional terms was due to the incorporation of databases that contain viral-specific annotation, such as ACLAME. Since VMGAP also performs additional analyses such as HMM, CDD and environmental DB searches as well as MeGO/GO and EC number assignments, it provides a more comprehensive repertoire of evidence types that may facilitate the discovery of novel viral functions as well as comparative analyses of metagenomic datasets.

Regarding the VMGAP implementation, the generation and storage of results into a relational sqlite database presents many advantages over working with flat files. The sqlite database allows the pipeline to monitor the status of each process launched on the grid and, in case of failure, restart the pipeline from the point that it crashed. Also, it makes it easier to query results, integrate different data types when generating summary reports, and share this information since all the analysis data (i.e. programs, parameters, cutoffs) and their results are stored in a single sqlite file. The storage of data in an sqlite database, however, might present some loading speed challenges when the data volume is very large and the speed of the storage device where the database resides is not fast enough (e.g. 7,200 RPM SATA drives). At JCVI, sqlite databases typically reside in 15,000 RPM SAS drives, with bandwidths of ~ 500 MB/sec. For slower systems, we recommend avoiding the usage of these databases and rather parse the results directly from the raw outputs of the analyses to generate the annotation and evidence files.

The organizational format of the output tab-delimited files, annotation and evidence are also advantageous. Since the first column of these files contains unique protein identifiers, all of the annotation and supporting evidence for any protein or group of proteins can be retrieved using the Unix grep utility directly from the command line. These files can be also imported into Excel for their inspection and analysis. Lastly, the VMGAP pipeline can be easily updated and customized to meet the specific needs and objectives of the user through the addition of additional virus-specific databases as they become available or the inclusion of more specialized boutique databases (e.g. RNA virus specific datasets) respectively.
